# „Palliative care“ bei Patienten mit Linksherzunterstützungssystem: systematische Übersichtsarbeit

**DOI:** 10.1007/s00101-021-00967-y

**Published:** 2021-04-30

**Authors:** T. Tenge, D. Schlieper, M. Schallenburger, S. Meier, J. Schwartz, M. Neukirchen

**Affiliations:** 1 Interdisziplinäres Zentrum für Palliativmedizin, Universitätsklinikum Düsseldorf, Medizinische Fakultät, Heinrich-Heine-Universität, Düsseldorf, Deutschland; 2 Klinik für Anästhesiologie, Universitätsklinikum Düsseldorf, Medizinische Fakultät, Heinrich-Heine-Universität, Düsseldorf, Deutschland; 3 Klinik für Anästhesiologie, Universitätsklinikum Düsseldorf, Moorenstraße 5, 40225 Düsseldorf, Deutschland

**Keywords:** Bridge to transplant, Destination therapy, Herzinsuffizienz, Trigger, Interdisziplinäre Zusammenarbeit, Bridge to transplant, Destination therapy, Heart failure, Trigger, Interdisciplinary cooperation

## Abstract

**Hintergrund:**

Bei terminal herzinsuffizienten Patienten gewinnt die Implantation von Linksherzunterstützungssystemen (LVAD) als Therapieoption zunehmend an Bedeutung. Diese Systeme werden als Überbrückung bis zu einer Herztransplantation (BTT) oder als definitive Therapie (DT) eingesetzt. Sie können die Lebensqualität verbessern und die Lebenszeit verlängern. Trotzdem bleibt die Prognose besonders bei DT oder bei Wechsel von BTT zu DT mit Blick auf die Lebenszeit und auftretende Komplikationen ungünstig. Bisher ist ungeklärt, ob eine LVAD-Implantation eine Indikation für eine frühzeitige Integration von Palliativmedizin darstellt.

**Ziel der Arbeit:**

Erfassung der aktuellen Studienlage über den Einfluss einer palliativmedizinischen Behandlung bei LVAD-Patienten.

**Material und Methoden:**

Im Mai 2020 wurde eine systematische Literaturrecherche in 6 verschiedenen Datenbanken durchgeführt.

**Ergebnisse:**

Von den 491 Treffern der Literaturrecherche wurden 21 Arbeiten in diese Übersichtsarbeit eingeschlossen. Durch die frühzeitige Integration der Palliativmedizin vor LVAD-Implantation erhöhte sich die Anzahl der Patienten mit vorausschauender Versorgungsplanung und Vorsorgeinstrumenten. Außerdem zeigte sich ein positiver Einfluss auf das familiäre Umfeld, das Symptommanagement und die Umstände des Versterbens. Es gibt verschiedene Formate für die Integration palliativmedizinischer Konzepte in die LVAD-Therapie.

**Diskussion:**

Die frühzeitige und kontinuierliche Einbindung der Palliativmedizin im Verlauf einer LVAD-Therapie kann die Behandlungsqualität verbessern. Die Ausarbeitung von spezifischen Handlungsempfehlungen ist in Abhängigkeit vom Therapieziel (BTT oder DT) sinnvoll. Empfohlen werden Schulungen für Palliativmediziner und LVAD-Spezialisten.

## Abstract (en)

**Background:**

An increasing number of patients reach the final stage of heart failure with heart transplantation as the only curative treatment. Mechanical circulatory support, such as left ventricular assist devices (LVAD) are becoming increasingly more important at this stage of the disease. An LVAD improves the quality of life and prolongs the lifespan. The LVAD is used as a bridge to transplantation (BTT) for patients waiting for a donor heart or as a destination therapy (DT) with no transplantation intended; nevertheless, implantation is often associated with complications and the prognosis remains unfavorable in DT cases or after transition from BTT to DT. For patients the device may be a source of physical and psychological distress and can become a burden for families and caregivers. So far it remains unclear whether LVAD treatment is an indication for concurrent palliative care.

**Objective:**

The aim of this study was to collect the current data on the influence of palliative care in LVAD patients and to identify possible formats of palliative care in clinical practice.

**Material and methods:**

In May 2020, a systematic literature search was performed using the PICOS instrument in six different databases, i.e. PubMed, Cochrane library, Google scholar, Scopus, Web of Science and Journals@Ovid. We included quantitative and qualitative studies in English and German. Case reports, comments and pediatric studies were excluded.

**Results:**

A total of 21 publications from an initial number of 491 were included in this review. The integration of palliative care in the context of LVAD implantation increased the number of advance directives and documented surrogate decision makers. Studies found a positive influence of palliative care on the conditions and place of death, family involvement and symptom and pain management. Involvement in the decision-making process may improve patient selection for LVAD treatment and helps patients to make the decision on informed consent. Various formats for the integration of palliative medical concepts into LVAD treatment are reported. It is not clear when palliative care involvement should start; however, most articles support an early and continuous integration in the LVAD process. We have aggregated possible topics for palliative care consultations from a number of publications. Mutual teaching of both LVAD and palliative care teams is recommended. Integration of a dedicated LVAD palliative care specialist as part of the LVAD team can support care for patients as well as clinicians.

**Conclusion:**

The role of palliative care in LVAD patients in clinical practice in the German-speaking area is not standardized. Early and continuous integration of palliative care into the course of LVAD treatment can improve the quality of care. Benefits for LVAD patients, caregivers and clinicians are described. For this purpose, recommendations as well as professional training for palliative care practitioners are useful. Further studies are needed to clarify the impact of palliative care in both DT and BTT patients.

Ein „left ventricular assist device“ (LVAD) wird als mechanisches linksventrikuläres Kreislaufunterstützungssystem zur Überbrückung bis zur Transplantation oder als definitive Therapie bei terminaler Herzinsuffizienz eingesetzt. Allerdings ist die Morbidität hoch und die Prognose besonders bei einer „destination therapy“ begrenzt. Bei anderen lebensbedrohlichen Erkrankungen mit eingeschränkter Lebenserwartung ist eine frühzeitige palliativmedizinische Mitbehandlung sinnvoll. Gilt das auch für Patienten mit LVAD? Diese systematische Übersichtsarbeit soll Antworten geben.

## Einleitung

Mechanische Kreislaufunterstützungssysteme wie „left ventricular assist devices“ (LVAD) können die Wartezeit bis zu einer Herztransplantation überbrücken („bridge to transplant“, BTT). Wenn eine Transplantation nicht (mehr) indiziert oder gewünscht ist, kann sie als definitive „destination therapy“ (DT) bis zum Versterben des Patienten eingesetzt werden.

In Deutschland nimmt die Zahl der LVAD-Implantationen kontinuierlich zu, 2018 waren es 903 Implantationen, gegenüber 390 im Jahr 2009 [3]. Die Gesamtletalität für das erste Jahr nach Implantation beträgt fast 20 % [13]. Vier Jahre nach Implantation sind ca. 50 % der LVAD-Patienten verstorben [13]. Insgesamt versterben die meisten Patienten im Krankenhaus [8]. Neben den physischen Belastungen vor und nach der Implantation kommt es häufig auch zu psychosozialen Belastungen [1].

Ebenso wie Patienten mit malignen Tumorerkrankungen profitieren herzinsuffiziente Patienten von einer palliativmedizinischen Versorgung [6]. Im Vergleich zu onkologischen Patienten werden diese Patienten jedoch seltener und später palliativmedizinisch mitbehandelt [14]. In der nationalen Versorgungsleitlinie zur chronischen Herzinsuffizienz wird bei terminaler Herzinsuffizienz eine nach Bedürfnissen und Symptomatik orientierte Integration von spezialisierter Palliativversorgung in komplexen Belastungssituationen befürwortet [2]. Bei unzureichender Datenlage wird hierfür allerdings nur eine abgeschwächte Empfehlung ausgesprochen [2]. Im Gegensatz zu den USA, wo „palliative care“ routinemäßig in die LVAD-Therapie integriert wird [9, 11], gibt es im deutschsprachigen Raum bislang keine Handlungsempfehlungen für die palliativmedizinische Mitbehandlung von terminal herzinsuffizienten LVAD-Patienten.

Ziel dieser Arbeit ist, anhand der aktuellen Datenlage zu überprüfen, ob die LVAD-Therapie eine Indikation für eine palliativmedizinische Mitbehandlung darstellt. Es ergeben sich folgende Fragestellungen:

1. Welche Auswirkungen hat eine palliativmedizinische Mitbehandlung auf klinisch messbare Endpunkte von LVAD-Patienten?
2. Wie könnte eine palliativmedizinische Mitbehandlung umgesetzt werden?
3. Gibt es unterschiedliche Empfehlungen zu DT und BTT?

## Methode

Die strukturierte Literaturrecherche erfolgte nach PICOS [26]:

- Population: LVAD-Patienten,
- Intervention: palliativmedizinische Mitbehandlung,
- Kontrollgruppe: keine,
- Ergebnisse: Bedarf, Nutzen und Folgen bei den Patienten sowie die Umsetzung der Mitbehandlung,
- Studienarten: quantitative und qualitative Studien sowie Übersichtsarbeiten.

**Figure 1:**
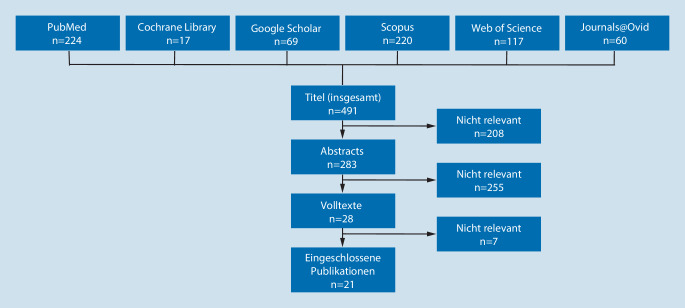

Im Mai 2020 wurde eine systematische Suche in 6 relevanten Datenbanken durchgeführt (Abb. 1). Die Suchstrategie lautete: ((heart-assist-device AND left) OR lvad OR Left-ventricular-assist* OR mechanical-circulatory-support OR Herz-Unterstützungs-System OR mechanisches-Kreislauf-Unterstützungssystem OR linksventrikuläres-Unterstützungssystem) AND (palliativ* OR advance-care-planning OR Terminal-Care OR End-of-life support OR Withdrawal). Eingeschlossen wurden Original- und Übersichtsarbeiten auf Englisch oder Deutsch seit 2008. Ausgeschlossen wurden Fallstudien, Kommentare und Arbeiten zu nichtlinksventrikulären Unterstützungssystemen oder mit rein pädiatrischem Fokus.

## Ergebnisse

In der Literaturrecherche konnten 21 relevante Volltexte identifiziert werden, die in Tab. 1 zusammengefasst sind.

**Table 1:** 

Quelle	Studiendesign	Probanden, Ort	Inhalte	Nur DT
Byram 2012 [4]	Nichtsystematische Übersicht	USA	Themen in Palliativkonsilen	✔
Chuzi et al. 2019 [5]	Retrospektive Datenanalyse	*n* = 68, USA	Ort, Zeitpunkt, Setting und Themen in präoperativen Palliativkonsilen	✔
Delmaczynska und Newham 2019 [7]	Systematische Übersicht	UK	Prävalenz und Folgen von „advance care planning“	–
Goldstein et al. 2011 [10]	Nichtsystematische Übersicht	USA	4 Zeitpunkte der Palliativintegration im Verlauf Kommunikationstechnik	–
Johnson und Kautz 2013 [12]	Nichtsystematische Übersicht	USA	Rolle der Pflegenden	✔
Luo et al. 2016 [15]	Nichtsystematische Übersicht	USA	Herausforderungen und Aufgaben im Verlauf Checkliste zum Deaktivieren des Linksherzunterstützungssystems (LVAD)	–
Nakagawa et al. 2017 [18]	Prospektive Beobachtung	*n* = 112, USA	Präoperative Palliativkonsile steigern das Familienbewusstsein für inakzeptable Gesundheitszustände „Bridge to trasplant“ (BTT): schwieriger, den inakzeptablen Gesundheitszustand zu nennen	–
Nakagawa et al. 2018 [16]	Retrospektive Datenanalyse	*n* = 89, USA	Palliativkonsile gleich häufig in „destination therapy“ (DT) und BTT, i. d. R. 4 im letzten Lebensmonat Mehr Tode außerhalb Intensivstationen, weniger lebenserhaltende Maßnahmen	–
Nakagawa et al. 2020 [17]	Prospektive Beobachtung	*n* = 72, USA	Einfluss einer Artikulation von Wünschen, Zielen und Limitationen im Palliativkonsil Patienten starben seltener auf der Intensivstation, häufiger im Hospiz, weniger Ethikkonferenzen Gleich häufig: lebenserhaltende Maßnahmen, Frequenz von Palliativkonsilen, LVAD-Deaktivierung	–
O’Connor et al. 2016 [19]	Prospektive Beobachtung	*n* = 37, USA	Skript für Pfleger Spezialisiertes Palliativkonsil nur bei unkontrollierten Symptomen, psychosozialem Stress und unklaren Pflegezielen Gutes *Triage*-System, Ressourcen- und Kostenersparnis möglich	✔
Sagin et al. 2016 [20]	Interviewbasierte qualitative Studie	*n* = 13, USA	Zusammenarbeit zwischen Palliativmedizin und LVAD-Team besonders durch Edukation gesteigert, spezialisierte LVAD-Palliativmediziner nützlich Aufgaben der Palliativmedizin Verbessertes Symptommanagement	✔
Salomon et al. 2018 [21]	Retrospektive Datenanalyse	*n* = 51, USA	Anstieg von Palliativkonsilen, besonders durch Teilnahme an wöchentlichen LVAD-Sitzungen Analyse von Inhalten in Palliativkonsilen Meist kein *Follow-up* notwendig, einzig signifikanter Prädiktor: Depression Palliativkonsil nie Grund von LVAD-Implantationsverzögerungen	–
Sinha et al. 2017 [22]	Retrospektive Datenanalyse	*n* = 122, USA	Vorsorgevollmachten gestiegen Gleich viele Tode im Krankenhaus	–
Slavin und Warraich 2020 [23]	Nichtsystematische Übersicht	USA	Darstellung und Bewertung verschiedener Zeitpunkte für die Palliativintegration	–
Swetz et al. 2011 [24]	Retrospektive Datenanalyse	*n* = 19, USA	Fallbeispiele Vermehrt Patientenverfügungen (PV) durch Palliativkonsile	✔
Swetz et al. 2014 [25]	Handlungsempfehlung	USA	Ausarbeiten eines *Preparedness-Plans* Idealer Zeitpunkt ist unklar, idealerweise präoperativ	–
Verdoorn et al. 2017 [27]	Retrospektive Datenanalyse	*n* = 107, USA	Anstieg der PV, nur 2 erwähnen LVAD	✔
Warraich et al. 2019 [28]	Nichtsystematische Übersicht	USA	Praktische Tipps für Palliativmediziner bezüglich LVAD Kommunikationstechnik zum besseren Verständnis Schulungen sinnvoll	–
Woodburn et al. 2019 [29]	Quantitative Studie mit Beobachtungen und Befragungen	*n* = 41 Patienten, *n* = 28 Betreuende, USA	Folgen der palliativmedizinischen Integration: erhöhte Lebensqualität, vermehrtes *Preparedness-Planning* sowie PV, Zufriedenheit der Ärzte	✔
Wordingham et al. 2017 [31]	Nichtsystematische Übersicht	USA	Aufgaben der Palliativmedizin Checkliste zur LVAD-Deaktivierung	–
Wordingham und McIlvennan 2019 [30]	Nichtsystematische Übersicht	USA	Aufgaben der Palliativmedizin	–

### Auswirkungen auf klinisch messbare Endpunkte

**Figure 2:**
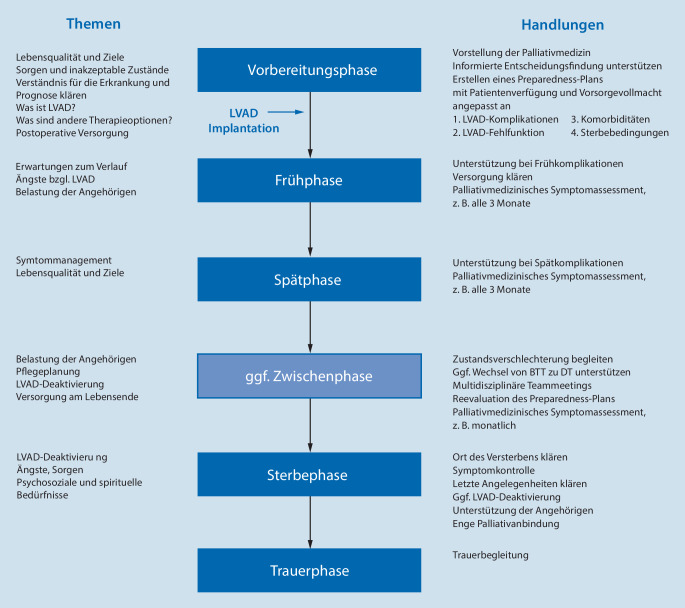

Eine palliativmedizinische Mitbehandlung erfolgt im Krankenhaus in der Regel durch ein konsiliarisch tätiges Palliativteam. In Studien, bei denen solche Palliativkonsile vor Implantation durchgeführt wurden, lagen anschließend signifikant mehr Patientenverfügungen (PV) und Vorsorgevollmachten (VV) vor [7, 22, 27, 30]. Der Anteil an PV mit spezifischem Bezug auf LVAD war allerdings auch nach Palliativkonsilen gering [15, 27]. Das Erstellen eines an den LVAD-Verlauf angepassten „Preparedness“-Plans mit PV und VV wird empfohlen (Abb. 2; [24, 25]). Im Verlauf der Therapie soll dieser dann gemäß den Wünschen und Sorgen der Patienten reevaluiert werden [15]. Durch Beteiligung von Angehörigen bei den präoperativen Gesprächen konnte deren Verständnis für die Wünsche der Patienten erhöht werden [18]. In diesen Gesprächen wurden Themen wie akzeptable Gesundheitszustände, LVAD-Deaktivierung und individuelle Präferenzen für die Sterbephase bereits früh angesprochen.

Wünsche mit Blick auf den Ort des Versterbens (Intensivstation oder zu Hause) konnten bei Vorliegen einer PV und nach durchgeführten Palliativkonsilen [7, 16, 17] häufiger umgesetzt werden. Unklarheit herrscht über den Einfluss palliativmedizinischer Einbindung auf lebenserhaltende Maßnahmen wie mechanische Beatmung und Nierenersatzverfahren sowie die Verlegungsrate in ein Hospiz [16, 17].

Die Integration der Palliativmedizin verbessert das Symptommanagement [20]. Ebenfalls wird die Lebensqualität von Patienten und den betreuenden Angehörigen erhöht [29], und die Zufriedenheit der primär behandelnden Ärzte steigt [29]. Auch zeigte sich ein Einfluss auf die Entscheidungsfindung: Nach einer „Shared-decision-making“*-*Intervention entschieden sich weniger Patienten für eine DT-LVAD-Implantation [23].

### Umsetzung der Mitbehandlung

Da eine Beratung durch ein Palliativteam gerade für die Therapiezielfindung von Bedeutung ist [5, 27], sollte die Einbindung idealerweise schon in der Vorbereitungsphase der Implantation beginnen [29]. In der Praxis findet der erste Kontakt zur Palliativmedizin allerdings häufig erst auf Intensivstationen kurz vor der Implantation [5] oder bei LVAD-Deaktivierung statt [30]. Insgesamt kommt es durch die palliativmedizinische Einbindung nicht zu einer Verzögerung der Implantation [21]. Das Krankenpflegepersonal, das bei der Versorgung von LVAD-Patienten eine wichtige Rolle spielt [12], kann bei der Identifikation von palliativmedizinischem Bedarf hilfreich sein [19] und eine frühzeitigere Einbindung der Palliativmedizin ermöglichen. Eine qualitative Analyse stellte den Nutzen speziell geschulter LVAD-Palliativmediziner, die kontinuierlich an den multidisziplinären LVAD-Sitzungen teilnahmen, heraus [20]. Auch die Bedeutung gegenseitiger Schulungen von LVAD-Spezialisten und Palliativmedizinern wurde betont [20, 28, 31]. Um Palliativmedizinern mehr Sicherheit im Umgang mit LVAD zu geben, wurden praxisnahe Ratschläge formuliert [28]. In Abb. 2 sind Themen- und Handlungsschwerpunkte für eine palliativmedizinische Begleitung bei DT-LVAD zusammengefasst.

### Unterschiedliche Empfehlungen zu DT und BTT

Die Implantation eines LVAD als DT ist zumindest aus palliativmedizinischer Sicht als eine palliative Maßnahme bei einer lebenszeitverkürzenden Erkrankung zu verstehen. Viele Studien (Tab. 1) und besonders die konkreten Empfehlungen (Abb. 2) beziehen sich daher explizit auf diese Patientengruppe. Aber auch für BTT-Patienten ist eine LVAD-Implantation eine Belastung, und die Letalität bleibt, obgleich geringer als bei DT-Patienten, bis zur Transplantation erhöht [13]. Eine Analyse zeigte ähnliche Sterbebedingungen bei BTT und DT [16]. Schließlich werden nicht alle BTT-Patienten tatsächlich transplantiert. Vor allem bei einer Therapiezieländerung von BTT nach DT kann eine palliativmedizinische Mitbehandlung hilfreich sein [16].

## Diskussion

Die vorliegenden Studien zeigen, dass eine palliativmedizinische Mitbehandlung positive Effekte für LVAD-Patienten, deren Angehörige und die behandelnden Ärzte hat und damit die Behandlungsqualität insgesamt verbessert wird [7, 15–24, 27, 29, 30]. Es konnten keine Arbeiten zum Einfluss auf Überleben, Komplikationsraten oder Komorbiditäten wie psychische Erkrankungen gefunden werden.

Obwohl über eine steigende Inanspruchnahme palliativmedizinischer Begleitung in der Sterbephase nach Einführung präoperativer Palliativkonsile in den USA [16] berichtet wird, war der Bedarf an Palliativkonsilen teilweise gering und nur bei gleichzeitigem Vorliegen einer Depression erhöht [21]. Insgesamt verdeutlichen konkrete Fallbeispiele den Nutzen der Palliativintegration [21]. Es zeigte sich, dass auch BTT-Patienten von palliativmedizinischer Begleitung profitieren können. Konkrete Empfehlungen zur palliativmedizinischen Begleitung bezogen sich eher auf DT-Patienten, trotzdem bleibt die Datenlage über den besten Zeitpunkt der Palliativintegration uneindeutig [25]. Eine kontinuierlich verfügbare Begleitung wird empfohlen [4]. Die Ausarbeitung von spezifischen Triggern, z. B. eine Therapiezieländerung oder aufgetretene Komplikationen, kann hilfreich sein, um den Bedarf für ein palliativmedizinisches Assessment besser zu bestimmen. Ebenfalls soll das Bewusstsein bei Kardiologen, Kardiochirurgen und Intensivmedizinern für den Nutzen einer palliativmedizinischen Behandlung bei terminal herzinsuffizienten (LVAD)-Patienten gestärkt werden. Bislang ist die klinische Versorgung herzinsuffizienter Patienten von einer „death-denying culture“ geprägt [6]. Auch für Palliativmediziner sind Schulungen über LVAD notwendig, um das Verständnis für die Indikationen und die Therapieprinzipien zu verbessern.

Fast alle eingeschlossenen Studien stammen aus Nordamerika. Daten zur Situation im europäischen oder im deutschsprachigen Raum liegen nicht vor.

Schließlich wären deutschsprachige Empfehlungen zur zeitgerechten palliativmedizinischen Unterstützung von LVAD-Patienten und deren Angehörigen wünschenswert.

## Fazit für die Praxis

- Die palliativmedizinische Mitbehandlung bei „left ventricular assist devices“ (LVAD) hat positive Effekte auf das Symptommanagement, die Lebensqualität von Patienten und Angehörigen sowie die Zufriedenheit von Ärzten.
- Die Vorsorgeplanung mit Patientenverfügungen und Vorsorgevollmachten wird erhöht.
- Eine Begleitung bereits im Entscheidungsprozess unterstützt eine partizipative Entscheidungsfindung.
- Der Sterbeort kann häufiger dem Patientenwunsch entsprechend außerhalb einer Intensivstation sein.
- Palliativmedizinische Integration wird noch nicht standardisiert durchgeführt; es fehlen evidenzbasierte spezifische Handlungsempfehlungen für Zeitpunkt, Ort und Rahmenbedingungen der Mitbehandlung.
- Weitere Analysen zum palliativen Bedarf in Abhängigkeit vom Therapieziel sind hilfreich.
- Gegenseitige Schulungen von LVAD-Spezialisten und Palliativmedizinern sind notwendig.
